# An Extract from Shrimp Processing By-Products Protects SH-SY5Y Cells from Neurotoxicity Induced by Aβ_25–35_

**DOI:** 10.3390/md15030083

**Published:** 2017-03-22

**Authors:** Yongping Zhang, Guangling Jiao, Cai Song, Shelly Gu, Richard E. Brown, Junzeng Zhang, Pingcheng Zhang, Jacques Gagnon, Steven Locke, Roumiana Stefanova, Claude Pelletier, Yi Zhang, Hongyu Lu

**Affiliations:** 1 Research Institute for Marine Drugs and Nutrition, College of Food Science and Technology, Guangdong Ocean University, Zhanjiang 524088, China; zhangyp2015@163.com (Y.Z.); hubeizhangyi@163.com (Y.Z.); irislhy@126.com (H.L.); 2 Department of Psychology and Neuroscience, Dalhousie University, Halifax, NS B3H 4R2, Canada; xiaomei.gu@dal.ca (S.G.); Richard.Brown@dal.ca (R.E.B.); pczhang2000@yahoo.com (P.Z.); 3 Coastal Zones Research Institute Inc., 232B, avenue de l’Église, Shippagan, NB E8S 1J2, Canada; sebrina2006@gmail.com (G.J.); jacques.gagnon@umoncton.ca (J.G.); Claude.pelletier@umoncton.ca (C.P.); 4 Aquatic and Crop Resource Development, National Research Council of Canada, 1411 Oxford Street, Halifax, NS B3H 3Z1, Canada; Junzeng.Zhang@nrc-cnrc.gc.ca (J.Z.); Roumiana.Stefanova@nrc-cnrc.gc.ca (R.S.); 5 Graduate Institute of Neural and Cognitive Sciences, China Medical University Hospital, Taichung 40402, Taiwan; 6 Aquatic and Crop Resource Development, National Research Council of Canada, 550 University Avenue, Charlottetown, PE C1A 4P3, Canada; Steven.Locke@nrc-cnrc.gc.ca

**Keywords:** Acetone extract from shrimp processing by-product (4-2A), polyunsaturated fatty acids, Aβ_25–35_, human neuroblastoma cell (SH-SY5Y), neuroprotection, Alzheimer’s disease (AD)

## Abstract

Increased evidence suggests that marine unsaturated fatty acids (FAs) can protect neurons from amyloid-β (Aβ)-induced neurodegeneration. Nuclear magnetic resonance (NMR), high performance liquid chromatography (HPLC) and gas chromatography (GC) assays showed that the acetone extract 4-2A obtained from shrimp *Pandalus borealis* industry processing wastes contained 67.19% monounsaturated FAs and 16.84% polyunsaturated FAs. The present study evaluated the anti-oxidative and anti-inflammatory effects of 4-2A in Aβ_25–35_-insulted differentiated SH-SY5Y cells. Cell viability and cytotoxicity were measured by using 3-(4,5-Dimethylthiazol-2-yl)-2,5-diphenyltetrazolium bromide (MTT) and lactate dehydrogenase (LDH) assays. Quantitative PCR and Western blotting were used to study the expression of neurotrophins, pro-inflammatory cytokines and apoptosis-related genes. Administration of 20 μM Aβ_25–35_ significantly reduced SH-SY5Y cell viability, the expression of nerve growth factor (NGF) and its tyrosine kinase TrkA receptor, as well as the level of glutathione, while increased reactive oxygen species (ROS), nitric oxide, tumor necrosis factor (TNF)-α, brain derived neurotrophic factor (BDNF) and its TrkB receptor. Aβ_25–35_ also increased the Bax/Bcl-2 ratio and Caspase-3 expression. Treatment with 4-2A significantly attenuated the Aβ_25–35_-induced changes in cell viability, ROS, GSH, NGF, TrkA, TNF-α, the Bax/Bcl-2 ratio and Caspase-3, except for nitric oxide, BDNF and TrKB. In conclusion, 4-2A effectively protected SH-SY5Y cells against Aβ-induced neuronal apoptosis/death by suppressing inflammation and oxidative stress and up-regulating NGF and TrKA expression.

## 1. Introduction

The neurodegenerative process in Alzheimer’s disease (AD) is associated with progressive accumulation of intracellular and extracellular neurotoxic amyloid-β (Aβ) oligomers in the brain [1,2,3]. Excessive Aβ-deposition may induce AD through oxidative stress and neuroinflammation. Amyloid-beta oligomers can activate microglia in vitro and in vivo [4], resulting in the production and release of reactive oxygen species (ROS) and pro-inflammatory cytokines such as tumor necrosis factor (TNF)-α, both of which can cause neural degeneration. Elevated levels of ROS interfere with the actions of many key molecules including enzymes, membrane lipids and DNA, which leads to cell apoptosis or death [5,6]. Increased pro-inflammatory cytokine release may stimulate neurons to produce increased amounts of Aβ oligomers and cause neuronal dysfunction and apoptosis [7,8]. Another hallmark of AD is decreased neurogenesis due to the dysfunction in neurotrophic signaling mechanisms [9]. In particular, nerve growth factor (NGF) and brain-derived neurotrophic factor (BDNF) and their receptors in the brain are disrupted. Reduced BDNF expression in the brain is a common feature of AD and cognitive dysfunction [10]. In addition, Aβ peptides are able to interfere with BDNF signal transduction pathways involved in neuronal survival and synaptic plasticity, hampering the transmission of neurotrophic responses [11].

Because the etiology of AD remain unknown, treatments that target AD are ineffective and often cause severe side-effects [12]. Most neurodegenerative diseases, including AD, are irreversible because the failure of neurogenesis and the increase in neuron death occurs before the clinical symptoms appear [13]. Thus, much effort is directed towards the discovery of neural pathways and their molecular mechanism that can be targeted by novel therapeutics to prevent AD. Natural substances with anti-oxidative and/or anti-inflammatory activity could provide effective treatments for the prevention of AD.

In the past decade, many studies have demonstrated that unsaturated fatty acids of marine origin, such as omega (*n*)-3 and *n*-9 fatty acids, could play a beneficial role in brain functions. Our previous studies have highlighted the effectiveness of dietary *n*-3 polyunsaturated fatty acids (PUFAs) as a potential treatment strategy for affective diseases [14,15]. Recent studies have demonstrated that eicosapentaenoic acid (EPA) and docosahexaenoic acid (DHA) possess neuroprotective properties because of their anti-oxidant and anti-inflammatory functions [14,15,16]. There are also data showing neuroprotective potential of monounsaturated fatty acids (MUFAs). For example, oleic acid, which belongs to *n*-9 MUFAs, has been found to modulate mitochondrial dysfunction, insulin resistance and inflammatory signaling [17,18,19] and act as neurotrophic factors for neurons [20]. Palmitoleic acid, a naturally occurring 16-carbon *n*-7 MUFA, and one of the most abundant fatty acids in the serum and tissues, was considered to be a lipokine and has been found to benefit some physiological function, such as regulating cell proliferation, and decreasing the expression of pro-inflammatory mediators and adipokines [21,22,23].

**Figure 1:**
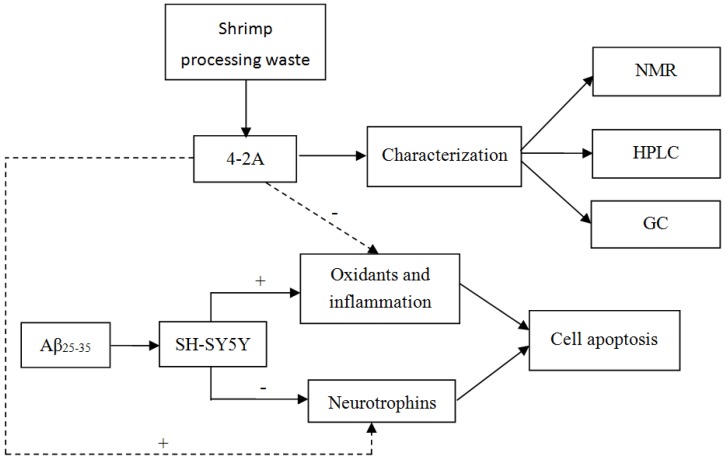
Experimental design and contents. NMR: Nuclear Magnetic Resonance; HPLC: High Performance Liquid Chromatography; GC: Gas Chromatograph. “+”: strengthening; “−”: weakening.

Tchoukanova and Benoit [24] developed a method to recover organic solids and oils from marine by-products. Using this method, shrimp oil was produced and found to be rich in long-chain unsaturated fatty acids [25]. The solid residue may also contain nutritional and bioactive ingredients that can be exploited. In the present study, an acetone extract 4-2A obtained from the solid phase was found to be rich in *n*-3 and *n*-9 unsaturated fatty acids. Because of the above mentioned neuroprotective function of unsaturated fatty acids, the present study aimed to determine whether the 4-2A extract from shrimp by-products would protect neurons from Aβ-induced neurotoxicity via its regulation of neurotrophic function, anti-inflammatory and anti-oxidative effects. To carry out this experiment, a cellular model of AD was set up using Aβ_25–35_-insulted differentiated SH-SY5Y cells. Following this, the effects of 4-2A on Aβ_25–35_-induced changes in cell viability, oxidative stress (ROS, NO, and GSH) and neurotrophins (NGF, BDNF and their TrkA and TrkB receptors) were measured. Since TNF-α is a key “pro-neuropathic” cytokine [26] and can activate a pro-apoptotic factor JNK pathway and trigger cellular death signaling [27], TNF-α expression was measured to test 4-2A anti-inflammatory effect in the present study. Then, the ability of 4-2A to regulate the expression of apoptosis-related genes (Bcl-2, Bax and Caspase-3) in the model was explored. The experimental design and content is presented in Figure 1.

## 2. Results

### 2.1. Characterization of Shrimp Extract 4-2A

**Figure 2:**
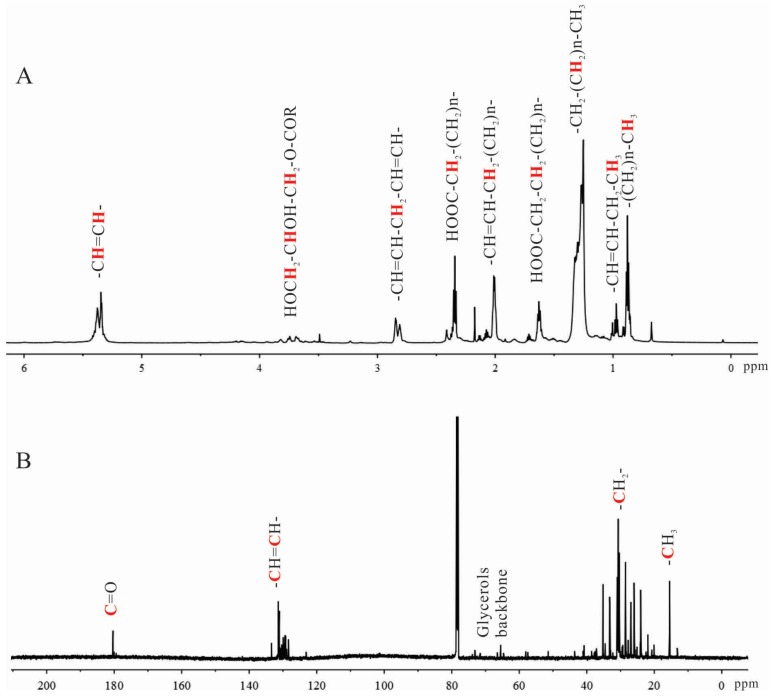
Characterization of 4-2A by NMR: (**A**) ^1^H-NMR spectrum (*n* ≥ 1); and (**B**) ^13^C-NMR spectrum.

Reddish and oily sample 4-2A was extracted from the solid residue of shrimp processing waste using acetone and yielded 2.55% of original dry mass. Both ^1^H-NMR and ^13^C-NMR spectra indicated that this shrimp extract was rich in lipids. The detailed assignments of the most common proton and carbon signals are shown in Figure 2. They have been compiled in the online resources of AOCS Lipid Library by a wide range of fatty compounds [28]. The spectra ranging from 0 to 6 ppm in the ^1^H-NMR spectrum covered the proton chemical shifts of most lipids (Figure 2A). To be noted, the signals around 2.8 ppm indicated the presence of methylenes between two double bonds –CH=CH-CH_2_-CH=CH-, a typical feature of polyunsaturated fatty acids. The shifts at 0.93–1.01 ppm suggested the protons of ω-3 terminal methyl groups. The intensity of these signals indicated that 4-2A contained relatively high level of ω-3 PUFAs. The chemical shifts between 3.5 and 4.0 ppm showed the presence of small amount of monoglycerides.

In the ^13^C-NMR spectrum (Figure 2B), the chemical shifts at 180.3 ppm indicated the carbons of C=O groups. The shifts around 130 ppm showed the two olefinic carbon atoms of double bonds. The carbons of the terminal methyl groups of lipids were identified by the chemical shifts at 15.3–15.6 ppm. Collectively, the major components of 4-2A were identified by NMR as lipids containing large amount of unsaturated fatty acids.

**Figure 3:**
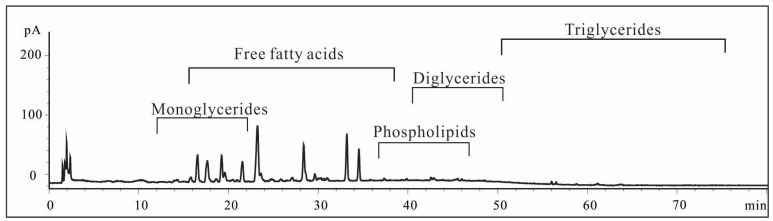
Characterization of 4-2A by HPLC with CAD detector.

Lipid standards were subjected on HPLC to qualify the separations. As indicated in Figure 3, the major components of 4-2A are free fatty acids and monoglycerides with elution window from 16 min to 36 min by charged aerosol detector (CAD). Based on the chromatogram, this extract does not contain much triglycerides and phospholipids. This is consistent with the result obtained from NMR analyses.

In order to understand the fatty acids profiles in this shrimp extract, the total fatty acids were released from 4-2A and analyzed on GC instrument. As a result shown in Table 1, the major FAME profiles of 4-2A was 18:1*n-*9 (20.65%), 16:1*n-*7 (14.75%), 22:1*n-*7 (11.04%), 20:5*n-*3 (8.45%), 20:1*n-*9 (7.97%), 18:1*n-*7 (7.07%), 16:0 (6.56%) and 22:6*n-*3 (6.54%). Most fatty acids (over 93%) are unsaturated, including 67.19% of monounsaturated fatty acids (MUFAs) and 16.84% of polyunsaturated fatty acids (PUFAs). To be mentioned, 4-2A contains 14.99% of omega-3 fatty acids (20:5*n-*3, Eicosapentaenoic acid, EPA and 22:6*n-*3, Docosahexaenoic acid, DHA).

In brief, the acetone extract 4-2A obtained from shrimp processing wastes was identified by NMR, HPLC and GC to contain mainly unsaturated free fatty acids and monoglycerides, including ω-3 PUFAs.

### 2.2. Aβ_25–35_-Induced Decrease in Neuronal Cell Viability Was Ameliorated by 4-2A

**Figure 4:**
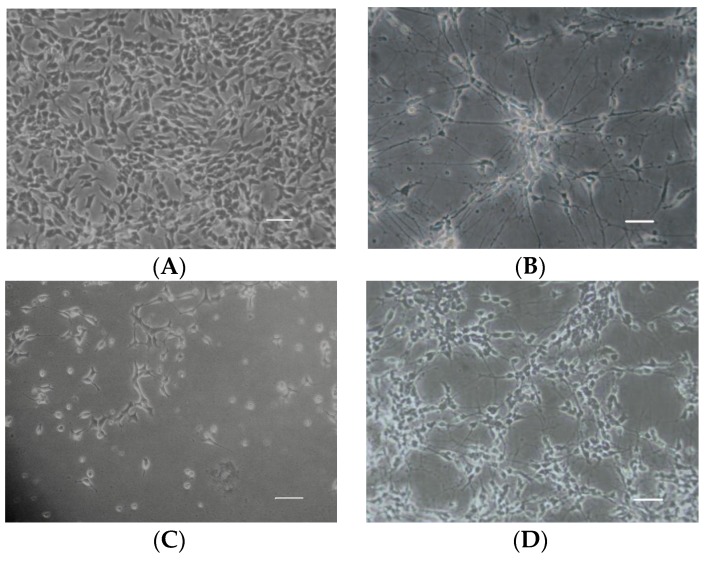
Cell morphology of undifferentiated SH-SY5Y cells (**A**); differentiated SH-SY5Y cells (**B**); differentiated SH-SY5Y cells insulted by Aβ_25–35_ (**C**); and differentiated SH-SY5Y cells treated with 4-2A and Aβ_25–35_ (**D**). Scale bar = 50 μm.

**Figure 5:**
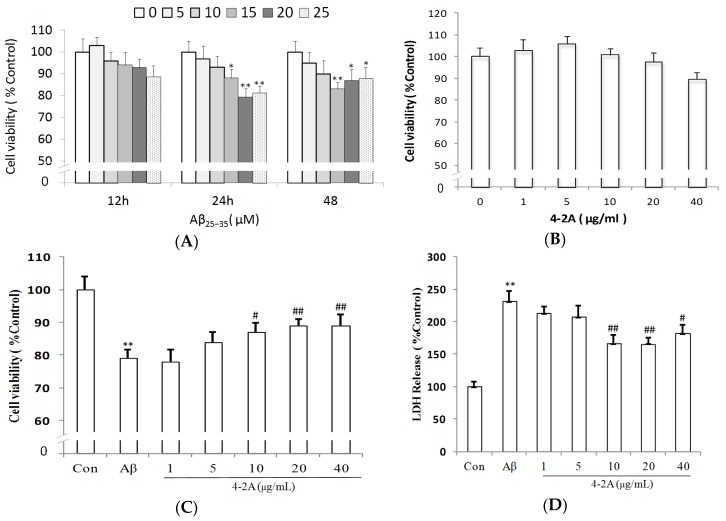
Cell viability and Cytotoxicity: (**A**) Treatment of Aβ_25–35_ at various doses for 12 h, 24 h and 48 h; (**B**) cells treated with 4-2A at indicated doses for 24 h; (**C**) changes in cell survival rate after the treatment with 20 μM Aβ_25–35_ for 24 h in the absence or presence of 4-2A at indicated doses; and (**D**) changes in lactate dehydrogenase (LDH) release after the treatment with 20 μM Aβ_25–35_ for 24 h in the absence or presence of 4-2A at indicated doses. Data represent mean ± SEM of three separate experiments (*n* = 4 in each experiment) and three mean values from independent experiment were used for statistics. * *p* < 0.05, ** *p* < 0.01 vs. control group; # *p* < 0.05, ## *p* < 0.01 vs. Aβ_25–35_ group.

The cell morphology of differentiated SH-SY5Y cells is more like the classic neuron-like cells with long synapse compared to undifferentiated cells (Figure 4). As detected by 3-(4,5-Dimethylthiazol-2-yl)-2,5-diphenyltetrazolium bromide (MTT) method, Aβ_25–35_ significantly decreased cell viability of differentiated SH-SY5Y cells in a time- and dose-dependent manner, such as at 15 μM for 12 h (*p* > 0.05), 24 h (*p* < 0.05) and 48 h (*p* < 0.01); and at 10 μM (*p* > 0.05), 15 μM (*p* < 0.05) and 20 μM (*p* < 0.01) for 24 h (Figure 5A). 4-2A treatment (1–20 μg/mL) alone did not exert any significant influence on the survival rate of SH-SY5Y cells, though 40 μg/mL of 4-2A slightly decreased the cell viability (*p* > 0.05) (Figure 5B). However, pretreated with different concentrations of 4-2A markedly attenuated the reduction of cell viability caused by Aβ_25–35_ in a dose-dependent manner at 1 (*p* > 0.05), 5 (*p* < 0.05), and 10–20 μg/mL (all *p* < 0.01) (Figure 5C). The protective role of 4-2A against Aβ_25–35_-induced insults in SH-SY5Y cells was further confirmed by lactate dehydrogenase (LDH) release assay (Figure 5D), which is an index of cell death. Combining the results from above assays, we could safely draw the conclusion that 4-2A could effectively protect the differentiated SH-SY5Y cells from Aβ_25–35_-induced cellular damage.

### 2.3. Treatment with 4-2A Attenuated the Changes in ROS, Nitric Oxide (NO) and Glutathione (GSH) Level Induced by Aβ_25–35_

**Figure 6:**
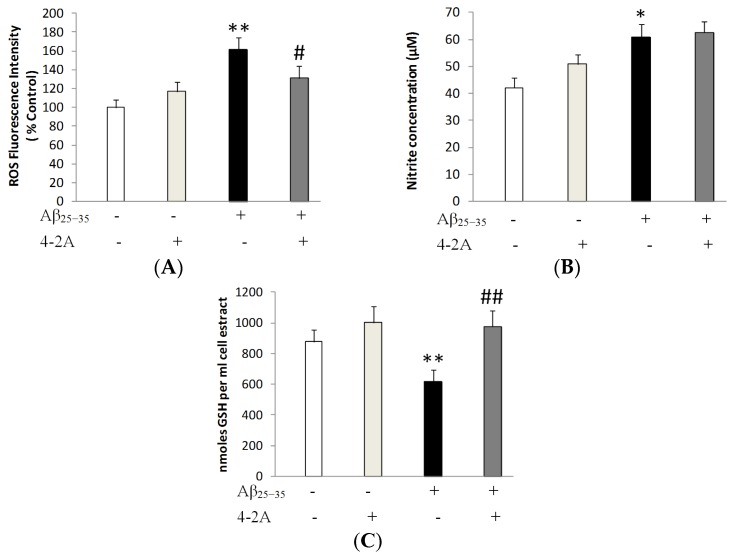
The effect of 4-2A on the changes of oxidative and anti-oxidative response induced by Aβ_25–35_. (**A**) ROS production in fully differentiated SH-SY5Y cells treated with Aβ_25–35_ 20 μM and/or 4-2A 10 μM for 24 h. Data represent mean ± SEM percentage of control. (**B**) NO production and (**C**) GSH content in fully differentiated SH-SY5Y cells treated with Aβ_25–35_ 20 μM and/or 4-2A 10 μM for 24 h. Data represent mean ± SEM of concentration (μM per mL). The statistics were based on data from six separate experiments, *n* = 6 in each experiment. * *p* < 0.05, ** *p* < 0.01 vs. control group; # *p* < 0.05, ## *p* < 0.01 vs. Aβ_25–35_ group.

Figure 6A illustrated that Aβ_25–35_ markedly increased ROS fluorescence when compared to control group (*p* < 0.01). However, cells pretreated with 4-2A (10 μg/mL) showed a partial decrease in mean fluorescence intensities by about 23% when compared to Aβ_25–35_-insulted group (*p* < 0.05). As shown in Figure 6C, a significant increase of about 44.42% in the level of nitrate was observed when the cells were treated with Aβ_25–35_ alone (*p* < 0.05), while 4-2A pre-treatment could not attenuate the Aβ_25–35_-induced change in NO concentration. The results shown in Figure 6C indicate a marked reduction in the GSH content of the Aβ_25–35_ insulted cells (*p* < 0.05), and 4-2A pre-treatment could completely restore this reduction (*p* < 0.01).

### 2.4. Treatment with 4-2A Attenuated the Aβ_25–35_-Inducued Increased Expression of Pro-Inflammatory Cytokine TNF-α

**Figure 7:**
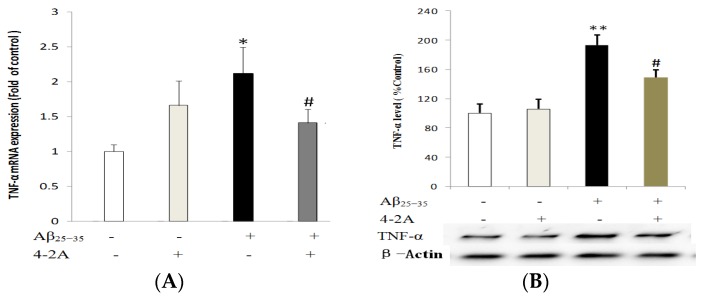
The expression of pro-inflammatory cytokine tumor necrosis factor-alpha (TNF-α). mRNA expression level (**A**); and protein expression level (**B**) of TNF-α, normalized to the corresponding level expression of housekeeping gene β-Actin, in differentiated SH-SY5Y cells treated with Aβ_25–35_ (20 μM) and/or 4-2A (10 μg/mL) for indicated times. The data of both mRNA and protein expression from six independent experiments (*n* = 1 in each experiment) were analyzed and expressed as mean ± SEM. * *p* < 0.05, ** *p* < 0.01 vs. control group; # *p* < 0.05 vs. Aβ_25–35_ group.

TNF-α mRNA expression was significantly increased by Aβ_25–35_ administration as early as 4 h of incubation (*p* < 0.05), but the increase in protein expression could not be found until 12 h of incubation with Aβ_25–35_ (*p* < 0.01). Pretreatment with 4-2A alone did not significantly affect TNF-α expression either in mRNA or in protein levels, but it could partially but significantly decrease the effect of Aβ_25–35_ (mRNA expression: *p* < 0.05 and protein expression *p* < 0.01, Figure 7).

### 2.5. Effect of 4-2A on the Expression of Neurotrophins: NGF and BDNF and Their TrkA and TrkB Receptors

**Figure 8:**
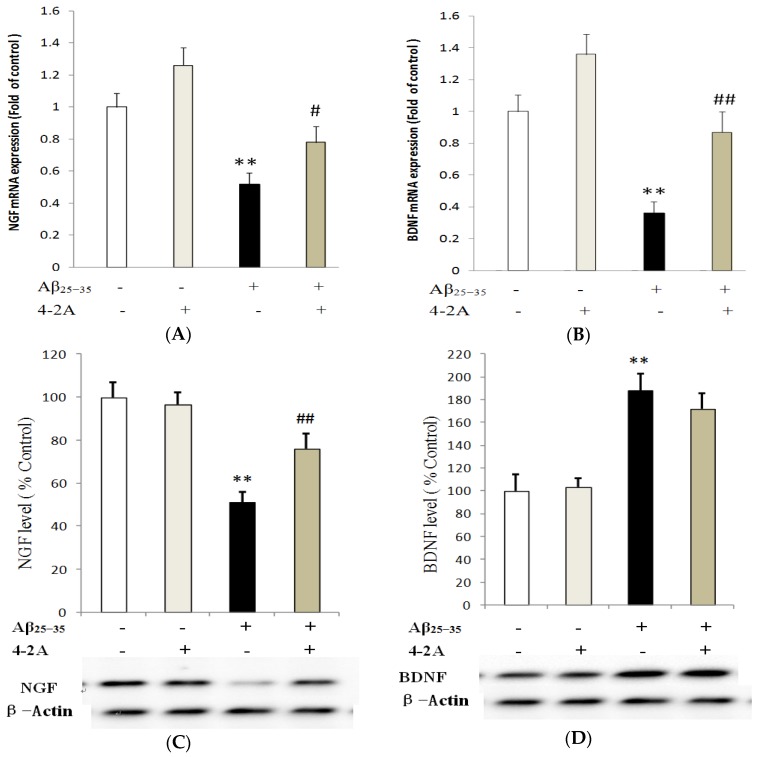
The effect of 4-2A on the expression of neurotrophin genes: NGF and BDNF. NGF mRNA expression (**A**); and protein expression (**C**); and BDNF mRNA expression (**B**); and protein expression (**D**), normalized to the expression of housekeeping gene β-Actin, in differentiated SH-SY5Y cells treated with Aβ_25–35_ (20 μM) and/or 4-2A (10 μg/mL) for 4, 8, 12, 24 h. The data of both mRNA and protein expression from six independent experiments (*n* = 1 in each experiment) were analyzed and expressed as mean ± SEM. ** *p* < 0.01 vs. control group; # *p* < 0.05, ## *p* < 0.01 vs. Aβ_25–35_ group.

Similar to TNF-α gene, the obvious change of NGF gene expression appeared at 4 h of Aβ_25–35_ treatment. Compared to control group, NGF mRNA expression in Aβ_25–35_-insulted cells was significantly down-regulated (*p* < 0.01, Figure 8A). Meanwhile, BDNF gene mRNA expression in Aβ_25–35_-insulted cells was also down-regulated at both 4 and 8 h (*p* < 0.01, Figure 8B). Compared to the control, a significantly decreased protein expression of NGF was found until 12 h incubation (*p* < 0.01, Figure 8C), whereas BDNF protein was significantly increased in Aβ_25–35_-insulted cells (*p* < 0.01, Figure 8D). However, in cells treated with 4-2A only, either NGF or BDNF gene was not affected at both mRNA and protein level compared to the control (*p* > 0.05). 4-2A pre-treatment could partially but significantly attenuate the Aβ_25–35_-induced change in NGF mRNA (*p* < 0.05) and protein (*p* < 0.01) expression (Figure 8A,C) and BDNF mRNA expression (both *p* < 0.01, Figure 8D), but not in BDNF protein expression (*p* > 0.05, Figure 8D).

**Figure 9:**
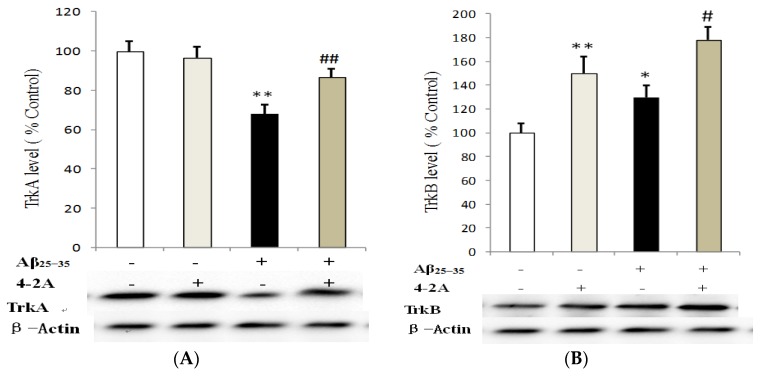
The effect of 4-2A on the expression of neurotrophin receptors: TrkA and TrkB. TrkA (**A**); and TrkB (**B**) protein expression, normalized to the protein expression of housekeeping gene β-Actin, in differentiated SH-SY5Y cells treated with Aβ_25–35_ (20 μM) and/or 4-2A (10 μg/mL) for 24 h. The data of protein expression from six independent experiments (*n* = 1 in each experiment) were analyzed and expressed as mean ± SEM. * *p* < 0.05, ** *p* < 0.01 vs. control group; # *p* < 0.05, ## *p* < 0.01 vs. Aβ_25–35_ group.

Unlike previously discussed genes, TrkA and TrkB were only affected at 24 h following Aβ_25–35_ administration. Aβ_25–35_ significantly decreased TrkA protein expression (*p* < 0.01). Pretreatment with 4-2A only had no effect on TrkA protein expression, but it could significantly attenuate the effect of Aβ_25–35_ on the receptor (*p* < 0.01, Figure 9A). In contrast to TrkA changes, TrkB protein in the cells treated with 4-2A only was significantly increased (*p* < 0.01) compared to the control. In the cells administrated with Aβ_25–35_ alone, a less but significantly increased protein of TrkB was also found (*p* < 0.05). 4-2A pretreatment did not reverse Aβ_25–35_-induced change, but further increased in TrkB expression (*p* < 0.05, Figure 9B).

### 2.6. Treatment with 4-2A Regulated the Expression of Apoptosis Related Genes: Bax, Bcl-2 and Caspase-3

**Figure 10:**
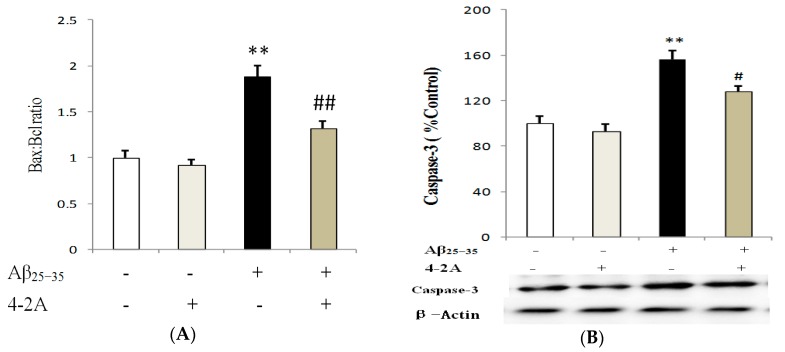
4-2A regulated the expression of apoptosis related genes: Bcl-2, Bax and Caspase-3. Bax:Bcl-2 ratio (**A**); and Caspase-3 (**B**) protein expression (normalized to the protein expression of housekeeping gene β-Actin) in differentiated SH-SY5Y cells treated with Aβ_25–35_ 20 μM and/or 4-2A 10 μg/mL for 24 h. The data of both mRNA and protein expression from six independent experiments (*n* = 1 in each experiment) were analyzed and expressed as mean ± SEM. ** *p* < 0.01 vs. control group; # *p* < 0.05, ## *p* < 0.01 vs. Aβ_25–35_ group.

The genes Bax, Bcl-2 and Caspase-3 protein expression were tested at different incubation times with Aβ_25–35_ (4, 8, 12 and 24 h). The expression of Bcl-2 was strongly decreased by Aβ_25–35_ at 24 h of incubation with Aβ_25–35_, which was significantly increased (*p* < 0.01) by 4-2A. 4-2A by itself had no effect on Bcl-2. With regard to Bax, no obvious change was found after 4–24 h incubation. The Bax: Bcl-2 ratio was significantly increased in cells administrated with Aβ_25–35_ alone (*p* < 0.01). Pretreatment with 4-2A significantly reversed the ratio of Bax: Bcl-2 to the control level (*p* < 0.01, Figure 10A). For Caspase-3, Aβ_25–35_ significantly increased its protein expression (*p* < 0.01), whereas pretreatment with 4-2A only did not affect the expression, but it could partially attenuate the effect of Aβ_25–35_ (*p* < 0.05, Figure 10B).

## 3. Discussion

Aβ_25–35_, an active fragment corresponding to amino acids 25–35 in full-length Aβ, possesses the same β-sheet structure and retains full toxicity of full-length Aβ_1–42_ [29]. Many experiments have demonstrated that Aβ_25–35_ can induce neurotoxicity and AD-like pathology, such as activating glial cells, increasing cholinesterase expression [30] and oxidative stress [31], as well as impairing spatial learning and memory [32,33]. Our previous study also showed that Aβ_25–35_ could result in neuroinflammatory response and dysfunction of neurotrophin system [34]. As such, Aβ_25–35_ has been popularly utilized to induce in vitro or in vivo AD models [32,33,35,36]. The present study demonstrates that 4-2A protects the SH-SY5Y cells from Aβ_25–35-_induced reduction in cell viability by suppressing oxygen stress and inflammation, regulating neurotrophin levels, hence attenuating neuron apoptosis.

Even though the exact pathophysiology of AD is unclear, oxidative stress has been found to play a fatal role in the pathogenic process of AD. Previous studies demonstrated that toxicity of Aβ_25–35_ in models of neurodegenerative diseases in vitro and in vivo was associated with the enhancement of ROS and NO liberation and oxidative damage [37,38,39,40,41], which up-regulated redox-sensitive transcription factors such as NF-κB, an important factor responsible for oxidative and inflammatory reactions in AD [42]. In agreement with these studies, Aβ_25–35_ increased the ROS and NO production from SH-SY5Y cells in the present study. ROS and NO are oxidants in the Alzheimer’s brain. However, NO is also a neurotransmitter, which may protect synapses by increasing neuronal excitability [43,44]. Thus, whether the Aβ-induced increase in NO acts as a compensatory and neuroprotective or neurotoxic role is unclear. The administration of 4-2A into Aβ_25–35_-treated cells partially but significantly decreased ROS production, which means 4-2A may partially protect neurons against free radicals or may improve mitochondrial dysfunction which is the major source of ROS. Furthermore, the decrease in GSH content caused by Aβ_25–35_ was significantly attenuated by 4-2A treatment. These findings indicated that 4-2A could restore the imbalance between oxidative stress factors and antioxidant systems. However, 4-2A could not affect the Aβ_25–35_-induced NO change, which may be related to a hypothesis that 4-2A may contribute to the self-protective ability of Aβ-insulted cells if this increase in NO is neuroprotective.

Inflammation is another important contributor to neurodegenerative diseases. Experimental and clinical findings provide evidence for the hypothesis that the neuronal degeneration in AD is not simply due to the Aβ deposition, but to neuroinflammation [45,46]. Consistent with the above studies, an increased expression of TNF-α gene was found in the present AD cellular model. Increased TNF-α can activate a pro-apoptotic factor JNK pathway that is involved in cell differentiation and proliferation [47] and trigger cellular death signaling [27]. In the present study, 4-2A showed anti-inflammatory property since it partially down-regulated the expression of TNF-α either in the mRNA or in protein level.

In our previous study, we showed that neuroinflammation could reduce the levels of neurotrophic factors, such as NGF [48] and BDNF [49]. In the present study, Aβ_25–35_ differently regulated the expression of the two neurotrophic factor genes. The decreased expression of NGF and its receptor TrkA seems to be reasonable for the low neuronal viability induced by Aβ. However, a significant increase in the expression of BDNF and its receptor TrkB protein in SH-SY5Y cells exposed to Aβ_25–35_ was unexpected, which was unparalleled by the decreased mRNA level in BDNF. The present data are partially in agreement with a previous in vitro study showing that the exposure of SH-SY5Y cells to Aβ_25–35_ induced a significant increase of BDNF [50]. We speculate that the increase of BDNF levels might act as a compensatory response against amyloid toxicity, while Aβ_25–35_ may trigger distinct effects on BDNF expression in different systems, conditions and incubation time.

Over-production of pro-inflammatory cytokines, oxidative stress and neurotrophin dysfunction may reduce neurogenesis and induce apoptosis [51,52], which result in neuronal death and memory loss in AD [53,54,55,56]. In the present study, Aβ_25–35_ treatment increased the pro-apoptotic Bax/anti-apoptotic Bcl-2 ratio and Caspase-3 expression, which were attenuated by pretreatment of 4-2A, suggesting 4-2A can regulate the imbalance between pro- and anti-apoptotic gene expression. The mechanism by which 4-2A attenuated Aβ-induced neuron damage may be through its unique components.

The acetone extract 4-2A from the shrimp by-products consists of lipids containing large amount of unsaturated fatty acids, especially monounsaturated fatty acid (MUFAs, 67.19%), including *n*-9 MUFAs and *n*-7 MUFAs. Oleic acid (18:1*n*-9) and palmitoleic acid (16:1*n*-7) are the most common MUFAs, which represent *n*-9 and *n*-7 MUFAs, respectively. The total fatty acids analysis profiles that 18:1*n-*9 (oleic acid, 20.65%) is the most abundant fatty acid in 4-2A. As the major *n*-9 MUFAs, oleic acid is high in olive oil which is the main characteristic of the Mediterranean Style Diet (MSD). Recent data from large epidemiological studies suggest a relationship between MSD adherence and significant reduction in incidence of Parkinson’s disease and AD and mild cognitive decline or risk of dementia [57]. Moreover, the protective effect of oleate against palmitate-induced mitochondrial dysfunction, insulin resistance and inflammatory signaling has been evaluated in several cell models [17,58]. Interestingly, in neuronal cells, Kwon et al. demonstrated that oleate preconditioning was superior to DHA or linoleate (18:2*n-*6) in the protection from the above palmitate-induced insults [58]. These effects may be associated with the neuroprotective ability of 4-2A to exert anti-inflammatory effects and restore the imbalance between oxidative stress factors and antioxidant systems. In addition, oleic acid may behave as a neurotrophic factor for neurons via up-regulation of molecular markers of axonal and dendritic growth, such as GAP-43 and MAP-2 [20]. This may be an important factor related to the ability of 4-2A to modulate Aβ_25–35_-induced abnormality in neurotrophic systems. These results strongly suggest that the ability of 4-2A to prevent SH-SY5Y cells from neurotoxicity induced by Aβ_25–35_ may be associated with its high content of oleic acid.

In recent years, *n*-7 palmitoleic acid (16:1*n*-7, 14.75%) has drawn increasing attention since its characterization as a bioactive lipid that coordinates metabolic crosstalk between the liver and adipose tissue [59]. Studies in cultured hepatocytes and mouse models of diet-induced obesity suggest that palmitoleic acid has anti-inflammatory and insulin-sensitizing effects [60]. Moreover, both in vitro and in vivo studies have demonstrated that palmitoleic acid can decrease the level of pro-inflammatory mediators and reduce the level of C-reactive protein in mice [21,23]. These anti-inflammatory effects may also contribute to the neuro-protective effect of 4-2A in the present study. However, because of rare reports about the effect of other MUFAs in health, the role of other two *n*-9 MUFAs (20:1*n*-9 and 22:1*n*-9) and three *n*-7 MUFAs (18:1*n*-7, 20:1*n*-7 and 22:1*n*-7) of longer chain length (≥C18) in 4-2A is unclear.

It is not surprising that 4-2A could protect SH-SY5Y neurons-like cells against Aβ_25–35_-induced apoptosis and death since 4-2A is rich in polyunsaturated *n*-3 fatty acids, such as EPA and DHA. Our previous studies have confirmed that *n*-3 fatty acids possess broad spectrum of neuroprotective activities in both in vitro and in vivo experiments because of their anti-oxidant and anti-inflammatory properties [61,62,63,64,65,66]. Additionally, Wu et al. [67] showed that *n*-3 PUFAs significantly attenuated the gene expression of pro-inflammatory cytokines, including IL-1β, IL-6, and TNF-α, and the protein levels of NF-κB and iNOS in brain tissues of rats with doxorubicin-induced depressive-like behaviors and neurotoxicity. In another in vivo study, Taepavarapruk and Song [48] revealed that *n*-3 PUFAs improved memory through IL-1-glucocorticoid-ACh release and IL-1-NGF-ACh release pathways. Moreover, there is evidence that dietary supplementation with DHA reduced the intraneuronal accumulation of not only amyloid-beta, but also tau, another important pathology marker for AD, in the 3xTg-AD mouse model via decreasing steady-state levels of presenilin 1 [68,69]. Based on these findings, there is no doubt that the neuroprotective effect of 4-2A was related to the rich *n*-3 PUFAs component. As the concentration of *n*-6 PUFAs are very low (1.85%), their effect in 4-2A can be ignored.

Finally, the saturated fatty acid in 4-2A may have no effect or only serve as an energy component in the cell cultural system because no biological activity was reported.

Taken together, shrimp processing by-product acetone extract 4-2A was prepared and characterized as lipids consisting of large amount of unsaturated fatty acids by NMR, HPLC-CAD and GC analyses. As a multifunctional agent, 4-2A showed potent inhibition against Aβ_25–35_ cytotoxicity, which confirms our hypothesis that 4-2A may exert neuroprotective effect via anti-oxidant, anti-inflammation and increasing neurotrophins. These effects of 4-2A may result from its various FAs components targeting various molecular pathways. The limitations of the present study are firstly that the treatment of 4-2A should be studied in the animal model of AD. Secondly, the effect of each FA component and their combination in different ratios should be determined, which can reveal the exact role of each FA and potential synergistic action of their combination in inflammatory, oxidant and neurotrophic functions in the brain. Thirdly, as the main components of neuronal membranes, the effect of FAs in 4-2A on the function of cell membrane and other mechanisms involved should also be investigated.

## 4. Materials and Methods

### 4.1. Preparation of the Shrimp Extract 4-2A

Fatty acids from the solid residue of shrimp *Pandalus borealis* processing waste were extracted with hexane (15 mL/g, *v*/*w* dry weight) at room temperature for 30 min, followed by 10 min of sonication, filtered, and then extracted with acetone under the same conditions. The liquid acetone extracts were combined, concentrated using rotary evaporator at reduced pressure, and then dried under N_2_. The resulting extract was named 4-2A.

### 4.2. Characterization of Shrimp Extract 4-2A

#### 4.2.1. Characterization of 4-2A by NMR

Both ^1^H-NMR and ^13^C-NMR spectra of 4-2A (1 mg, dissolved in 700 µL of 99.8% CDCl_3_) were recorded at 4 °C on a Bruker AV-III 700 MHz spectrometer (Bruker BioSpin Canada, Milton, ON, Canada), equipped with a 5 mm TCI cryoprobe. The data were processed using the standard TopSpin V 2.1 (Bruker BioSpin Canada, Milton, ON, Canada) software.

#### 4.2.2. Characterization of 4-2A by HPLC

HPLC analysis was carried out on an 1100 series instrument (Agilent Technologies, Santa Clara, CA, USA) with a Thermo Scientific™ Dionex™ Corona™ Charged Aerosol Detector (Burlington, ON, Canada). The method was adopted from dionex.com [70] with modifications. Samples were prepared by diluting 1 mg of analyte in 1 mL of methanol/chloroform (1:1, *v*/*v*). Ten microliter of each sample solution was injected on a HALO C8 column (2.1 mm × 100 mm, 2.7 μm, Advanced Materials Technology Inc., Wilmington, DE, USA) with a 0.45 mL/min flow rate at 40 °C. A 5 × 2.1 mm C8 cartridge (2.7 μm, Advanced Materials Technology Inc.) was used as a pre-column. Mobile phase A methanol/water/acetic acid (750:250:4) and phase B acetonitrile/methanol/tetrahydrofuran/acetic acid (500:375:125:4) were proceeded from 100% A at the beginning at 0.8 mL/min to 30% A and 70% B at 40 min at 1.0 mL/min, then reached to 20% A and 80% B in 10 min at 1.0 mL/min and finally to 100% B in 10 min at 1.0 mL/min, held for 10 min, and then back to 100% A in 10 min at 0.8 mL/min. The column temperature was at 40 °C.

Free fatty acids (myristic acid, *cis*-palmitoleic acid, *cis*-vaccenic acid, *trans*-vaccenic acid, oleic acid, elaidic acid, pentadecanoic acid, palmitic acid, heptadecanoic acid, *cis*-10-heptadecenoic acid, stearic acid, 9-*cis*,12-*cis*-linoleic acid, *cis*,*cis*,*cis*-9,12,15-octadecatrienoic acid, *cis*,*cis*,*cis*-6,9,12-octadecatrienoic acid, *cis*-11-eicosenoic acid, arachidonic acid, *cis*-5,8,11,14,17-eicosapentaenoic acid, *cis*-4,7,10,13,16,19-docosahexaenoic acid and tricosanoic acid), monoglycerides (dl-α-palmitin, 1-monopalmitoleoyl-*rac*-glycerol and 1-Oleoyl-*rac*-glycerol), diglycerides (1,2-distearoyl-*rac*-glycerol and 1,3-distearoylglycerol), triglycerides (olive oil), and phospholipids mixer (l-α-lysophosphatidylcholine, l-α-phosphatidylcholine, l-α-phosphatidylethanolamine and l-α-phosphatidy- linositol sodium salt) purchased from Sigma-Aldrich Inc. (St. Louis, MO, USA) were applied on the column under the same conditions.

#### 4.2.3. GC Analysis of 4-2A

GC analysis was performed as described by Jiao et al. [25]. Total fatty acids were released from 4-2A by hydrolyzing with 1.5 N NaOH methanol solution under N_2_ at 100 °C for 5 min, then methylated with 14% of BF_3_ methanol solution at 100 °C for 30 min. Distilled water was then added to stop the reaction. The methylated fatty acids were extracted with hexane and subjected on an Agilent Technologies 7890A GC spectrometer (Agilent Technologies, Santa Clara, CA, USA) using an Omegawax 250 fused silica capillary column (30 m × 0.25 mm × 0.25 μm film thickness, Sigma-Aldrich, St. Louis, MO, USA). Supelco^®^ 37 component fatty acid methylated esters (FAME) mix and PUFA-3 (Supelco, Bellefonte, PA, USA) were used as FAME standards.

### 4.3. SH-SY5Y Culture and Differentiation

SH-SY5Y cells were obtained from ATCC (CRL-2266, Lot. 61983120) and were maintained in DMEM/F12 medium (Gibco^®^, Burlington, ON, Canada) supplemented with 10% fetal bovine serum (FBS, Gibco^®^, Burlington, ON, Canada) and 1% penicillin–streptomycin in a humid atmosphere of 5% CO_2_ at 37 °C. SH-SY5Y cells were differentiated into fully human neuron-like cells with treatment all-*trans*-retinoic acid (RA, Sigma Aldrich, Oakville, ON, Canada), at a final concentration of 10 μM in DMEM/F12 with 3% FBS (media changed every 2 days), for 7–8 days.

### 4.4. Experimental Design

Once the SH-SY5Y cells were differentiated into classic neuron-like cells with long axons compared to undifferentiated cells, they were used in the experiments. Aβ_25–35_ (synthetic, ≥97% HPLC, Sigma Aldrich, Oakville, ON, Canada, A4559) was dissolved in sterile double-distilled water at a concentration of 1 mmol/L stock solution, was aged at 37 °C for 4 day, and then stored at −20 °C before use (This aging procedure was proved to produce birefringent fibril-like structures, globular amorphous aggregates and induce cognitive impairment in rats [34,71]). The 4-2A was dissolved in ethanol at a stock concentration of 100 mg/mL, and then added to DMEM/F12 (final ethanol concentration 0.1%). The effects of Aβ_25–35_ at 5, 10, 15, 20 and 25 μM at 12, 24 and 48 h and the effects of 4-2A at 1, 5, 10, 20 and 40 μg/mL on Aβ_25–35_-induced SH-SY5Y cell injury were then determined separately. The optimal dose and culture duration of Aβ_25–35_ (20 μM, 24 h) and 4-2A (10 μg/mL, pretreated for 12 h before addition of 20 μM Aβ_25–35_) were selected based on when the significant decrease in cell viability or attenuation of this effect appeared respectively. In the present study, four groups of SH-SY5Y cells were used: (i) control (cells in culture media which contained 0.1% *v*/*v* ethanol); (ii) 4-2A (cells in culture media with 10 μg/mL 4-2A; (iii) Aβ_25–35_ (cells in culture media with 20 μM Aβ_25–35_); and (iv) 4-2A and Aβ_25–35_ (cells pretreated with 10 μg/mL 4-2A for 12 h before addition of 20 μM Aβ_25–35_). After the addition of Aβ_25–35_, the SH-SY5Y cells were incubated for 24 h, after which cell viability, oxidative stress, inflammatory cytokine TNF-a, neurotrophins and apoptosis were measured.

### 4.5. Measurement of Cell Viability by MTT Assay

Cell viability was measured using MTT assay which measures the cell proliferation rate and the reduction in cell viability. Cells were seeded in 96-well plates and 90 µL of cell suspension added to each well. Following experimental treatment, 10 µL MTT (ATCC) was added to each well and the plate was incubated for additional 4 h at 37 °C. The optical density was measured at 570 nm using a microplate reader (BioTek, Winooski, VT, USA). The absorbance of the control group was considered as 100% of the cell viability.

### 4.6. Cytotoxicity Assay by LDH Assay

As MTT assay was sensitive to cell numbers which was affected by both cell proliferation and cell viability, it was essential to use another assay to confirm the result. The CytoTox-96 assay kit (Promega, Madison, WI, USA) was employed to evaluate the total release of cytoplasmic lactate dehydrogenase (LDH) into the medium, which is a consequence of cellular integrity damage. The assay is based upon a coupled enzymatic conversion from 2-*p*-(iodophenyl)-3-(*p*-nitrophenyl)-5-phenyltetrazolium chloride (INT, a tetrazolium salt) into a formazan product, and the enzymatic reaction is catalyzed by LDH released from cells and diaphorase in the assay substrate mixture. Absorbance was read at 490 nm by the microplate reader. The mean absorbance of each group was normalized to the percentage of the control value.

### 4.7. Measurement of Oxidative Stress and Antioxidant Response

The intracellular level of ROS was measured with a fluorometric intracellular ROS kit (Sigma Aldrich) according to manufacturer’s instructions. The fluorescence intensity was detected at lex = 650/lem = 675 nm using a fluorescence microplate reader (Reader Synergy HT, BioTek). The intracellular level of NO production was determined by the Griess Reagent System (Promega) according to manufacturer’s instructions. The absorbance was measured at 540 nm using a microplate reader.

The level of GSH was determined with a glutathione assay kit (Sigma Aldrich) according to manufacturer’s instructions. The fluorescence intensity was measured by a fluorimeter plate reader set at an excitation wavelength of 390 nm and emission wavelength of 478 nm.

### 4.8. Determination of Gene Expression with Quantitative PCR

SH-SY5Y cells treated as described above were harvested. The total RNA was extracted as recommended by the manufacturer (RNeasy^®^ Lipid Tissue Handbook, Qiagen, Germantown, MD, USA). Complementary DNA (cDNA) was synthesized from 2 μg RNA using the GoScript™ Reverse Transcriptase (Promega, Madison, WI, USA). Primer sequences (Table 2) were obtained from Invitrogen Corporation. PCR reactions were prepared using Quantitect SYBR Green master mix (Qiagen, Germantown, MD, USA) and carried out using a Real-Time PCR Detection Systems (Bio-Rad, Hercules, CA, USA) CFX96™ Real-Time System. The real-time PCR was optimized to run with conditions of the initial incubation at 95 °C for 5 min, denaturation at 94 °C for 15 s, annealing at 59 °C for 30 s, and extension at 72 °C for 30 s with a single fluorescence measurement and up to 38 cycles. Expression levels of target mRNAs were normalized to beta actin (relative quantification) with the ΔΔCT correction.

### 4.9. Analysis of Protein Expression by Western Blotting

SH-SY5Y cells treated as described above were collected and centrifuged at 10,000× *g* for 10 min. The cell pellet was lysed with a RIPA buffer (RIPA, Thermo Fisher Scientific, Hudson, NH, USA) aided by sonication, and then spun down at 10,000× *g* for 10 min at 4 °C. The supernatant was collected and aliquots containing 20–40 μg of protein were loaded after boiling and separated on 10% SDS PAGE gels at 100 V for 60 min in electrophoresis buffer. After running the gels, the proteins were transferred to Polyvinylidene difluoride (PVDF) membranes (Millipore, Bellerica, MA, USA). The membranes were blocked with 5% non-fat milk in Tris buffer saline for 1 h at 20 °C. Following blocking, blots were washed with TBST for 5 min and incubated with primary antibodies, including the rabbit origin polyclonal antibodies for Actin (Abcam, Cambridge, MA, USA; ab8227, 42 kDa, 1:4000), NGF (Abcam, ab52918, 27 kDa, 1:500), BDNF (Abcam, ab6201, 28kDa, matured form, 1:200), TrkA (Abcam, ab59272, 85 kDa, 1:800), TrkB (Thermo Fisher Science, Hudson, NH, USA; MA5-14903, 90-140 kDa, 1:1000), TNF-α (Abcam, ab9739, 17 kDa, 1:2500), Bcl-2 (Abcam, ab136285, 26 kDa, 1:5000), Bax (Abcam, ab32503, 21 kDa, 1:2000) and Caspase-3 (Abcam, ab44976, 32 kDa, activated forms, 1:500), overnight at 4 °C, followed by the secondary antibody, peroxidase (HRP)-conjugated anti-rabbit IgG (Abcam, ab6721, 1:5000), for 1 h at 20 °C. The blots were washed in TBS three times. Immunoreactive bands were detected by Clarity™ Western ECL Substrate Kit (Bio-Rad, Hercules, CA, USA) on a ChemiDoc™ MP System with Image Lab™ Software (Bio-Rad, Hercules, CA, USA). All target proteins were quantified by normalizing them to β-Actin re-probed on the same membrane and then calculated as a percentage of the control group.

### 4.10. Statistics

Results were expressed as mean ± SEM. Statistical evaluation was performed with IBM SPSS Statistics 22. For the dose–response test of 4-2A/Aβ_25–35_, results were analyzed by one-way ANOVA, followed by Dunnett post-hoc test in case of significant main effects (*p* < 0.05). The possible interaction between Aβ_25–35_ and 4-2A was measured by two-way ANOVA with Tukey’s post-hoc tests. A value of *p* < 0.05 was considered statistically significant.

## Figures and Tables

**Table 1:** Major fatty acid methylated esters (FAME) profiles of 4-2A by GC analysis.

FAME	Intensity (%)	Stdev (*n* = 4, %)
14:0	2.10	0.25
16:0	6.56	0.58
18:0	0.70	0.08
16:1*n-*7	14.75	0.21
18:1*n-*5	0.77	0.16
18:1*n-*7	7.07	0.31
18:1*n-*9	20.65	2.62
20:1*n-*7	1.08	0.03
20:1*n-*9	7.97	0.97
20:1*n-*11	1.95	0.01
22:1*n-*7	11.04	0.24
22:1*n-*9	1.91	0.78
18:2*n-*6	0.86	0.10
20:4*n-*6	0.99	0.12
20:5*n-*3	8.45	0.83
22:6*n-*3	6.54	1.02
Total	93.39	
Saturated	9.36	
Monounsaturated	67.19	
Polyunsaturated	16.84	
Omega-3	14.99	
Omega-6	1.85	
Omega-7	33.94	
Omega-9	30.53	

**Table 2:** Primer names and sequences.

Primer Names	Sequences
hActin F	GATGAGATTGGCATGGCTTT
hActin R	CACCTTCACCGTTCCAGTTT
hBAX F	GGGGACGAACTGGACAGTAA
hBAX R	CAGTTGAAGTTGCCGTCAGA
hBCL 2 F	TCTAGGGGAGGTGGTAGGCT
hBCL 2 R	CTGAGCAAGTCAGAGACCCC
hCaspase 3 F	GACTCTAGACGGCATCCAGC
hCaspase 3 R	TGACAGCCAGTGAGACTTGG
hNGF F	ACCTTTCTCAGTAGCGGCAA
hNGF R	TGTGTCACCTTGTCAGGGAA
hTNF-α F	AGGTTTGGCCTCACAAGGAC
hTNF-α R	GCGGTAGGGACAGTTCACAG
hBDNF F	GGAGACACATCCAGCAAT
hBDNF R	ACAAGAACGAACACAACAG
hTrkB F	CTATGCTGTGGTGGTGATT
hTrkB R	CCGAAGAAGATGGAGTGTTA
hTrkA F	TACAGCACCGACTATTACC
hTrkA R	ATGATGGCGTAGACCTCT
